# Gait Speed among Older Participants Enrolled in an Evidence-Based Fall Risk Reduction Program: A Subgroup Analysis

**DOI:** 10.3389/fpubh.2015.00026

**Published:** 2015-04-27

**Authors:** Jinmyoung Cho, Matthew Lee Smith, Tiffany E. Shubert, Luohua Jiang, SangNam Ahn, Marcia G. Ory

**Affiliations:** ^1^Center for Applied Health Research, Baylor Scott & White Health, Temple, TX, USA; ^2^Department of Health Promotion and Community Health Science, Texas A&M Health Science Center School of Public Health, College Station, TX, USA; ^3^Department of Health Promotion and Behavior, The University of Georgia, Athens, GA, USA; ^4^School of Medicine, University of North Carolina, Chapel Hill, NC, USA; ^5^Department of Epidemiology, School of Medicine, University of California, Irvine, CA, USA; ^6^Department of Epidemiology and Biostatistics, Texas A&M Health Science Center School of Public Health, College Station, TX, USA; ^7^Division of Health Systems Management and Policy, The University of Memphis, Memphis, TN, USA

**Keywords:** older adults, A Matter of Balance/Volunteer Lay Leader model, timed up and go

## Abstract

**Background:**

Functional decline is a primary risk factor for institutionalization and mortality among older adults. Although community-based fall risk reduction programs have been widely disseminated, little is known about their impact on gait speed, a key indicator of functional performance. Changes in functional performance between baseline and post-intervention were examined by means of timed up and go (TUG), a standardized functional assessment test administered to participants enrolled in A Matter of Balance/Volunteer Lay Leader (AMOB/VLL) model, an evidence-based fall risk reduction program.

**Methods:**

This study included 71 participants enrolled in an AMOB/VLL program in the Brazos Valley and South Plain regions of Texas. Paired *t*-tests were employed to assess program effects on gait speed at baseline and post-intervention for all participants and by subgroups of age, sex, living status, delivery sites, and self-rated health. The Bonferroni correction was applied to adjust inflated Type I error rate associated with performing multiple *t*-tests, for which *p*-values <0.0042 (i.e., 0.5/12 comparisons) were deemed statistically significant.

**Results:**

Overall, gait speed of enrolled participants improved from baseline to post-intervention (*t* = 3.22, *p* = 0.002). Significant changes in TUG scores were observed among participants who lived with others (*t* = 4.45, *p* < 0.001), rated their health as excellent, very good, or good (*t* = 3.05, *p* = 0.003), and attended program workshops at senior centers (*t* = 3.52, *p* = 0.003).

**Conclusion:**

Findings suggest community-based fall risk reduction programs can improve gait speed for older adults. More translational research is needed to understand factors related to the effectiveness of fall risk reduction programs in various populations and settings.

## Falls among Older Adults

Falls among older adults are a serious public health problem in America (1). Approximately one-fourth of older adults aged 80 years and older experience at least two falls per year (2–4). As the risk of falling increases with advanced age, dramatic escalations in fall-related morbidity, hospitalization, institutionalization, and mortality can be expected to accompany the aging of the population (5). Direct annual medical care costs related to falls have been estimated at almost $20 billion and are projected to increase sharply in the coming decades (6, 7).

Various demographic, functional, and health factors are known to increase the risk for falling among older adults (8). These factors include age (2, 4), being female (9, 10), a prior history of falls (2, 4), gait and mobility deficits (2, 4, 9, 11), and poor self-reported health status (9, 10). In addition to personal characteristics, particular attention has been paid to the environmental circumstances surrounding falls, such neighborhood environments or program delivery settings (12).

Fall-prevention programs and integration of prevention services have been shown to decrease fall recurrence (13) and reduce health-care costs (14). However, literature about the effectiveness of evidence-based fall-prevention programs for improving objectively measured functional performance has been limited for programs delivered in the community by lay facilitators. Given its ease of use and standardization, gait speed, often called “timed up and go (TUG),” has been frequently used to assess functional performance as an outcome measurement for effective interventions (15, 16). Many studies have demonstrated a strong relationship between gait speed and fall-related risk, health and functional status, institutionalization, and mortality among older adults (17–19). To address the existing research gaps, the overall goal of this study was to examine improvement in functional performance among older participants enrolled in A Matter of Balance/Volunteer Lay Leader (AMOB/VLL) model, an evidence-based fall risk reduction program.

## A Matter of Balance/Volunteer Lay Leader Fall Risk Reduction Program

A Matter of Balance (AMOB), established at the Roybal Center for Enhancement of Late-Life Function at Boston University, is an evidence-based program to reduce risk of falls among older adults (20). The effectiveness of the AMOB program was originally tested through a randomized clinical trial, which showed positive improvements in physical activity and mobility control (21). Derived from the original program, the AMOB/VLL model has been adapted for widespread community dissemination in various health and aging sectors (22, 23). Delivered by trained lay-led facilitators in 38 states, it is presented in 2-h sessions for 8 weeks. One hour is taught by a physical therapist. This hour focuses on the role of exercise in fall prevention. It is not meant to be a stand-alone session, but rather an introduction for older adults to build upon. At the end of AMOB, participants are more likely to exercise and intended activity (21). Each session includes specific goals for older adults to reduce the risk of falling and continue remaining active and independent (24). The major goals of the program are as follows: to make participants perceive control, to increase their confidence, and to learn falls are controllable. The design of intervention targets behavior change and emphasizes building fall self-efficacy and setting goals for increasing physical activity through lectures, group discussions, various problem-solving and role-playing activities, exercise and assertiveness training, and individual assignments (24).

Since 2006, a well-established infrastructure has facilitated the delivery of the intervention to older adults in Texas (24). The network for aging services arranged a signed agreement with the Texas Association of Area Agencies on Aging (AAA) for implementation of the AMOB/VLL program in many AAA regions. The program targets low-income older minority adults and their caregivers and focuses on reaching a diverse population in a large geographic area. Residential facilities, health-care institutions, public health departments, faith-based organizations, business sectors, and local government were included as partners with the Texas AAA sites to build fall-prevention capacity (24, 25).

## Purpose of Study

**Figure 1:**
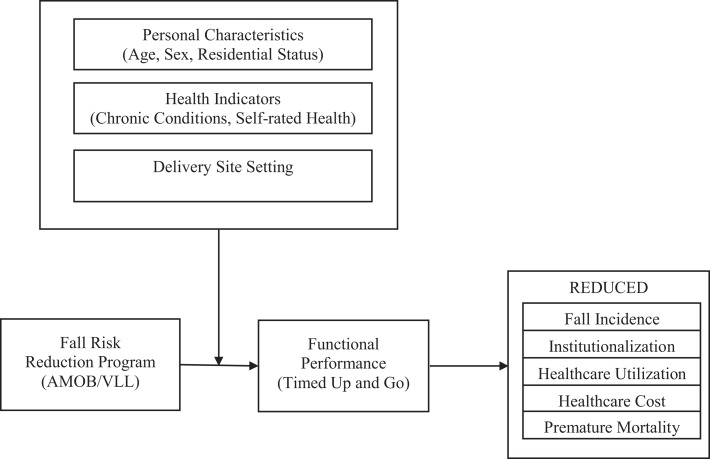
**Conceptual model**.

Although community-based health promotion programs result in for improvement in falls efficacy, overall health status, and increased physical activities (11, 26), less is known about their impact on physical performance (i.e., TUG) among older participants. The purposes of this study were to (a) assess the changes in physical performance measured by the TUG test from baseline to post-intervention and (b) compare the improvement in physical performance by personal characteristics and delivery sites. A conceptual model for this study is shown in Figure 1. This model depicts the fall risk reduction program as an intervention that can have positive effects on changes in physical performance. If participation in AMOB results in improved efficacy as well as improved physical performance, physical therapists may want to include AMOB as a program for appropriate patients. To better understand who may benefit the most from this program, personal characteristics and delivery sites act as moderators between the fall risk reduction program (as illustrated by the AMOB/VLL model) and physical performance as measured by the TUG test. In turn, the TUG measurements are associated with long-term improvements in reduced health-care use and costs as well as enhanced health and well-being.

## Methods

### Procedures and participants

**Figure 2:**
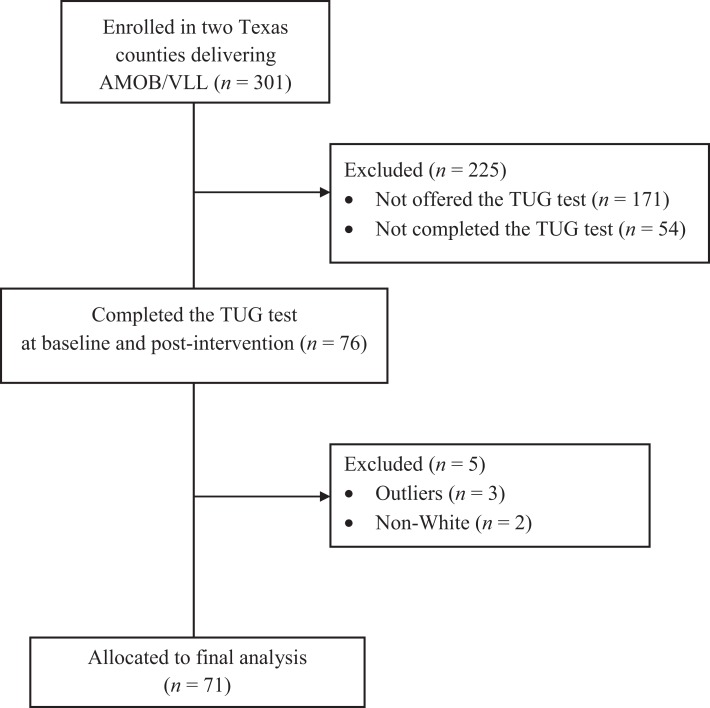
**Diagram for study participants inclusion**.

A total of 301 participants enrolled in the Texas AMOB/VLL fall risk reduction program between September 2007 and April 2009 in two regional AAAs: Brazos Valley and South Plain. Although functional assessment was optional in the statewide AAA delivery of the AMOB/VLL program, assessments were conducted in some classes that taught these two regions. Workshop leaders were trained in assessment procedures and performed assessments in eight of the AMOB/VLL classes, which served as the basis for this study. A total of 171 participants who attended classes in these two regions but who were not assessed using the TUG were excluded; thus, 76 participants completed the TUG test at baseline and post-intervention, whereas 54 participants did not complete the test at both times. Boxplots were used to screen for outliers for TUG scores from both baseline and post-intervention. Results indicated the presence of three outliers, who were then omitted. An additional two cases reporting an “other” ethnic group were excluded to maximize racial and ethnic homogeneity of participants for this study. As a result, only non-Hispanic White participants were included in this study. Figure 2 shows the recruitment flow diagram, indicating that 71 participants were included in study analyses.

### Measures

#### Timed up and go test

The TUG test, introduced in 1991 by Podsiadlo and Richardson (27), has been used extensively for over a decade to predict fall risk and to examine functional mobility among older adults (26, 28). It assesses the time in seconds that participants required to “rise from a standard arm chair, walk at your typical or normal pace to a line on the floor 3 meters away, turn, return, and sit down again” (p. 64) (27). This test was validated to test physical functioning and mobility among community-dwelling older adults (26) and showed high predictive validity with the Berg Balance Scale (27). Those who completed the TUG tasks in more than 14 s also showed lower scores on the Berg Balance Scale, which was associated with higher risk for institutionalization (26).

#### Personal characteristics

*Age* was coded as a continuous variable based on a participant’s birth date and ranged from 56 to 95. The age was then categorized into three groups for the purpose of the study: young-old (up to 69 years), mid-old (from 70 to 79 years), and old-old (80 years and older). *Sex* was scored 0 if the participant was male and 1 if the participant was female. *Living status* was scored 1 if participants lived with others and 0 if they lived alone. *Self-rated health* was included. Self-rated health, a single item measuring in which participants rate current status of their overall health, has been widely used as a significant predictor of physical and psychological health such as mortality or functionality among various populations (29–32). Many studies have shown that the single item is a reliable and valid measure reflecting objective health status (e.g., cardio-cerebral vascular diseases, visual impairment) (32–34). At baseline, participants were also asked to self-report their perceived health status: “Would you say that in general your health is poor, fair, good, very good, or excellent?” For comparisons of self-rated health, the responses were divided into two categories (i.e., poor/fair vs. good/very good/excellent).

#### Delivery sites

To compare outcomes at the various settings in which the AMOB/VLL program was conducted in the Brazos Valley and South Plain regions, delivery site types were obtained from administrative data. Delivery site categories included senior centers, community centers, faith-based organizations, residential facilities, and other Parks Department facilities. For comparisons of delivery sites, five sites were categorized into three groups: senior centers and community centers, residential facilities, and others.

#### Data analysis

The paired *t*-test was employed to compare mean TUG scores for all participants pre- and post-intervention. Statistical significance was examined at the level of 0.05 for this test. Then, a series of paired *t*-tests were employed to compare the TUG scores by subgroups: age groups, sex, residential status, delivery sites, and self-rated health. Bonferroni’s correction was applied for subgroup (12 groups) comparisons to adjust the inflated Type I error rate associated with performing multiple *t*-tests, for which *p*-values <0.0042 (i.e., 0.5/12 comparisons) were deemed statistically significant. Statistical analyses were conducted with SPSS statistical software (version 20.0). As an indicator of practical significance, Cohen’s *d* standardized effect sizes were calculated to compare intervention effects from baseline to post-intervention within each group.

## Results

### Sample characteristics

Table 1 summarizes characteristics of study participants. The average age of the study participants was 77.8 (SD = 9.3) years old. The majority of participants was female (80.6%), and more than half the participants lived with others (56.1%). Over three quarters of the participants rated their health good, very good, or excellent (75.4%). Most participants had at least one chronic health problem (84.4%). Within the two regional AAAs, the AMOB/VLL program was implemented in residential facilities (52.1%), senior centers (21.1%), faith-based organizations (12.7%), other Parks Department facilities (11.3%), and community centers (2.8%).

**Table 1:** **Characteristic of the study participants**.

Characteristics	Frequency (%)
Age, mean (SD) (range: 56−95)	77.8 (9.3)
Sex
Male	13 (19.4)
Female	54 (80.6)
Residential status
Living alone	29 (43.9)
Living with others	37 (56.1)
Self-rated health
Excellent	5 (7.7)
Very good	17 (26.2)
Good	27 (41.5)
Fair	15 (23.1)
Poor	1 (1.5)
Numbers of chronic condition
None	11 (15.5)
1–2	44 (61.9)
3+	16 (22.5)
Delivery sites
Senior centers	15 (21.1)
Community centers	2 (2.8)
Residential facilities	37 (52.1)
Faith-based organizations	9 (12.7)
Other-parks department facilities	8 (11.3)

*Different numbers of missing cases were observed for each variable. Missing cases were excluded from calculations and analyses*.

### Changes in timed up and go test

Before the paired *t*-test for the TUG score was conducted, the TUG scores at baseline and post-intervention were observed. Almost a third of participants (28.2%) at baseline and 22.5% of participants at post-intervention performed slower than 14 s, which represents a critical value on the TUG test. Table 2 presents results of the paired *t*-tests for TUG scores among all AMOB/VLL program participants and by subgroups from baseline to post-intervention. Among all participants, the average TUG score at baseline was 12.89 (SD = 5.08) and changed to 11.95 (SD = 4.30) at post-intervention (*t* = 3.22, *p* = 0.002). When comparing TUG score changes by subgroup, three significant improvements were found. First, participants who lived with others showed significant changes in TUG scores from baseline (*M* = 12.61, SD = 5.92) to post-intervention (*M* = 11.32, SD = 5.04), *t* = 4.45, *p* < 0.001. The effect size (Cohen’s *d*) was 0.23. Second, participants who attended the AMOB/VLL program at senior centers or community centers showed statistically significant improvement in TUG scores from 14.96 (SD = 7.20) at baseline to 13.30 (SD = 6.21) at post-intervention, *t* = 3.52, *p* = 0.003. Cohen’s *d* was 0.25. Third, those who perceived their health good, very good, or excellent showed significant improvement in TUG scores: 12.77 (SD = 5.41) at baseline and 11.87 (SD = 4.56) at post-intervention, *t* = 3.05, *p* = 0.003. The effect size was 0.18.

**Table 2:** **Average TUG scores in pre- and post-test by groups**.

	Pre (SD)	Post (SD)	*t*-Value	*p*	Cohen’s *d*
Total participants	12.89 (5.08)	11.95 (4.30)	3.22	0.002	0.25
Age groups
Young-old (*n* = 14)	9.74 (2.21)	8.89 (2.06)	2.60	0.018	0.40
Mid-old (*n* = 16)	11.67 (3.43)	10.83 (2.80)	2.25	0.040	0.27
Old-old (*n* = 34)	14.85 (6.06)	13.82 (5.00)	2.20	0.035	0.19
Sex
Male (*n* = 13)	12.41 (4.00)	11.53 (3.59)	1.33	0.208	0.23
Female (*n* = 54)	12.95 (5.45)	12.08 (4.62)	2.77	0.008	0.17
Living status
Living alone (*n* = 29)	12.93 (4.05)	12.66 (3.44)	0.52	0.605	0.07
Living with others (*n* = 37)	12.61 (5.92)	11.32 (5.04)	4.45	<0.001	0.23
Delivery sites
Senior/community centers (*n* = 15)	14.96 (7.20)	13.30 (6.21)	3.52	0.003	0.25
Residential facilities (*n* = 38)	13.58 (4.51)	12.79 (3.69)	1.56	0.128	0.19
Others (*n* = 19)	9.90 (2.27)	9.24 (2.02)	2.54	0.020	0.31
Self-rated health groups
Excellent/VG/good (*n* = 57)	12.77 (5.41)	11.87 (4.56)	3.05	0.003	0.18
Fair/poor (*n* = 11)	13.08 (2.78)	11.86 (3.36)	1.25	0.240	0.40

*Different numbers of missing cases were observed for each variable. Missing cases were excluded from calculations and analyses*.

## Discussion

The primary objective in this study was to examine changes in functional performance between baseline and post-intervention among participants enrolled in the Texas AMOB/VLL fall risk reduction program. Several important findings emerged from this study. First, the average score for all participants’ walking speed assessed with the standardized TUG test improved from baseline to post-intervention. These findings demonstrate that this fall risk reduction program can improve gait speed among old participants in addition to its previously reported benefits for falls efficacy and fear of falling (22). Second, subgroup comparisons showed significant improvements among those who rated their health more positively, lived with others, and attended program workshops in senior centers or community centers. These findings reveal that improvement in functional performance (i.e., TUG) may be directly associated with participating in a fall risk reduction program for these subgroups.

The most significant aspect of this study was the incorporation of an objectively measured functional assessment to compare participant improvement based on self-reported measures. Because most measures from evidence-based programs have been based on self-reported information, such as health-related quality of life, number of falls, and number of chronic conditions within the previous week or month, a couple of other researchers noted that self-reported measures might produce recall bias as a data collection limitation (23, 35, 36). Using a standardized functional assessment test (i.e., TUG) can contribute to the validation of previous findings that reported improvements in the ability to perform important social and role functions (23, 36).

Findings of the current study also highlight the importance of physical health, social, and environmental correlates to enhance the effectiveness of the evidence-based program. First, the analyses revealed that better perception of health was associated with significant improvement on the TUG test. It is obvious that those who perceived their health to be of better status showed significant improvement because these individuals may be more likely to have fewer chronic conditions and may be less influenced by daily activity limitations. However, the largest standardized Cohen’s effect size (Cohen’s *d* = 0.40) was notably observed among participants who self-reported their health to be fair/poor despite the lack of statistical significance of the TUG score change. In other words, those with worse health status may show larger changes in functional assessments because they have greater opportunity for improvement, whereas those healthier participants who score high at baseline have little room for improvement (36). This finding points to the need for future research to increase understanding of the functional improvements of individuals of different health status levels and detect underlying statistical effects, such as regression to the mean.

Second, the significant improvement in gait speed based on residential status emphasizes the importance of social correlates on the effectiveness of the evidence-based program. Results showed significant improvement in functional performance from baseline to post-intervention among participants who live with others. This finding is consistent with previous studies that has shown the significant relationship between physical activities and support from family or friends (37, 38). Living with others is likely to prevent older adults from social isolation, which has been identified as a barrier to physical activity (38). This finding may also indicate that participants who lived with others had social support mechanisms that may have encouraged them to attend more AMOB/VLL program sessions (i.e., received more intervention dose) and engage in recommended physical activities outside class time.

Third, findings of this study suggest delivery settings in which evidence-based programs that are implemented can enhance physical performance among old participants. Participants who attended workshops in senior centers or community centers showed significant improvement in TUG scores from baseline to post-intervention, which may highlight an environmental benefit for delivering evidence-based programs to older adults in these group settings. This finding may be associated with the notion that these participants were healthier upon program enrollment or that the location of the delivery site was more accessible, which increased their attendance (i.e., intervention does) and led to significant improvement.

In an attempt to disseminate widely fall-prevention programs, the Texas AAA sites have continued to build fall-prevention capacity by partnering with the public health network and others to establish programs in various settings, such as residential facilities, faith-based organizations, workplace setting, health-care institutions, public health departments, and governmental facilities (25). Although other studies have identified differences in program outcomes by delivery site types (35), further investigation is warranted to understand the influence of delivery site on functional assessment measures among lay-led fall-prevention programs.

### Limitations and implications

The findings of this study showed significant TUG score changes associated with this fall risk reduction program; however, a few notable limitations were associated with this study. First, this study included only 71 study participants. The small sample size may limit generalization of our findings to other populations. Second, as stated in the procedures and participant section, older minority adults were excluded from this study because too few participated for meaningful analyses. Although Texas is a geographically large and demographically diverse state, the two minority group cases were intentionally excluded to yield a homogeneous sample of participants. If enough minorities had been available for meaningful comparisons, we may find ethnic difference in functional performance among more diverse groups of participants. Finally, if objective method of rating current health status (e.g., biomarkers) was used in the fall risk reduction program instead of self-rated health, the result may provide an association between health status and functional capacity among old participants.

The findings from the current study have considerable implications for future research on translational studies. Although this study provides an important view of the use of TUG tests in a community-based fall risk reduction program, additional research is needed to link functional assessment scores to the actual fall experience, subsequent health-care use, and the availability of supportive environments illustrated in our conceptual model of fall risk behaviors, interventions, and long-term outcomes. First, the capacity for objective functional measurement among community-dwelling older adults should be built into evidence-based fall-prevention programs. For example, instructor manuals for lay leaders should include a training session about objective functional measurement. Such provider training is important for maintaining measurement necessary for research assessment. In recognition of the importance of objective measurements for purposes of both research and programing, this type of training has been built into CDC’s State Fall Prevention Program (39). Furthermore, future studies should focus on participants’ degree of disability to examine more comprehensively the effectiveness of evidence-based fall risk reduction programs in different populations. Considering the extent of the disability or investigating the difference in physical performance between fallers and non-fallers may suggest detailed strategies to promote physical activity for older adults with various baseline functional levels. Also, more translational research is needed to understand potential modifiable and non-modifiable correlates related to effectiveness of fall risk reduction programs on functional performance within various populations and settings.

## Conflict of Interest Statement

The authors declare that the research was conducted in the absence of any commercial or financial relationships that could be construed as a potential conflict of interest.

This paper is included in the Research Topic, “Evidence-Based Programming for Older Adults.” This Research Topic received partial funding from multiple government and private organizations/agencies; however, the views, findings, and conclusions in these articles are those of the authors and do not necessarily represent the official position of these organizations/agencies. All papers published in the Research Topic received peer review from members of the Frontiers in Public Health (Public Health Education and Promotion section) panel of Review Editors. Because this Research Topic represents work closely associated with a nationwide evidence-based movement in the US, many of the authors and/or Review Editors may have worked together previously in some fashion. Review Editors were purposively selected based on their expertise with evaluation and/or evidence-based programming for older adults. Review Editors were independent of named authors on any given article published in this volume.
